# Transcriptome Analysis of Muscle Growth-Related circRNA in the Pacific Abalone *Haliotis discus hanna*

**DOI:** 10.3390/genes16010065

**Published:** 2025-01-08

**Authors:** Jianfang Huang, Jian He, Zhenghan She, Mingcan Zhou, Dongchang Li, Jianming Chen, Caihuan Ke

**Affiliations:** 1 Fujian Key Laboratory on Conservation and Sustainable Utilization of Marine Biodiversity, Fuzhou Institute of Oceanography, College of Geography and Oceanography, Minjiang University, Fuzhou 350108, China; jianfhuang@mju.edu.cn (J.H.); hejian@mju.edu.cn (J.H.); 19835557185@163.com (Z.S.); 2 College of Ocean and Earth Sciences, Xiamen University, Xiamen 361102, China; 22320200155965@stu.xmu.edu.cn; 3 Jinjiang Fuda Abalone Aquaculture Co., Ltd., Quanzhou 362251, China; fudabaoyu@126.com

**Keywords:** abalone, *Haliotis discus hannai*, circRNA, growth, shellfish

## Abstract

(1) Background: Animal growth is a complex process, involving the coordination of a wide variety of genes, non-coding RNAs, and pathways. Circular RNAs (circRNAs) belong to a novel class of functional non-coding RNAs (ncRNAs). They have a distinctive ring structure and are involved in various biological processes, including the proliferation, differentiation, and apoptosis of muscle cells. The Pacific abalone *Haliotis discus hannai* is an economically valuable mollusk species cultivated in China. However, the modulation of muscle growth by circRNAs in this species is poorly understood. (2) Methods: In this study, we analyzed the muscle transcriptomes of 6 *H. discus hannai* specimens: three small (S_HD) and three large (L_HD) groups via RNA-seq and bioinformatics technology. (3) Results: The results indicated the presence of 11,744 circRNAs in abalone adductor muscle. Furthermore, the L_HD group had 250 significantly differentially expressed circRNAs (106 upregulated and 144 downregulated) relative to the S_HD group. Moreover, the bioinformatics assessment revealed that circRNAs were related to lipid transporter activity, lipid biosynthetic process, fat digestion and absorption, the single-organism metabolic process, the thyroid hormone signaling pathway, and the hippo signaling pathway, which regulates growth. Seventeen key candidate circRNAs were identified, and a core functional circRNA-miRNA-mRNA network associated with abalone muscle growth was described. Gene expression was verified using qRT-PCR, confirming the accuracy of the RNA-seq data. (4) Conclusion: Overall, this investigation furnishes novel evidence for the potential muscle growth modulatory mechanisms in Pacific abalone. These high-quality circRNA data of abalone muscle provide a reference for functional studies on the abalone genome.

## 1. Introduction

Circular RNAs (circRNAs) have a covalently closed-loop structure and are non-coding RNA (ncRNA) molecules. They were first discovered in the mid-1970s in hepatitis delta virus and plant viroids [1,2]. Until 2010, they were considered a result of experimental flaws or the byproduct of errors in endogenous RNA splicing [3]. However, circRNA became a research hotspot after the development of novel computational methods and biochemical techniques for RNA purification, which allowed their de novo identification in RNA sequencing data of non-model and model organisms [4,5,6]. The synthesis of circRNA initiates during pre-mRNA splicing, which is commonly coupled to RNA polymerase II-mediated transcription [7,8]. Moreover, they are produced by back-splicing, during which the 5′ end of an upstream splice site ligates to the 3′ end of a downstream splice site. CircRNAs are classified into three types: intronic, exonic, and exon-intronic circRNAs.

Several studies have indicated that circRNAs have specific spatial and temporal expression profiles in different tissues, and many are evolutionarily conserved [9,10]. Furthermore, their expression pattern, stability, and conservation have revealed that circRNAs have essential physiological and molecular modulatory roles [11]. Recent data suggest that mRNA is the target for various ncRNAs and miRNAs and modulates miRNA-mediated gene expression. Moreover, miRNAs can also target mRNA, lncRNA, circRNA, and pseudogenes. Competitive endogenous RNAs (ceRNAs) are those RNAs that compete for the same miRNA binding site. The most common regulatory function of circRNAs is their role as miRNA sponges, which has been comprehensively studied across the species. It has been observed that circRNAs have complementary sequences in microRNAs, which when recognized competitively deactivates them. Zhou et al. [12] indicated that the has-circ-0034326/miR-25-3p/*ITGA5* network influences the progression of hepatocellular carcinoma.

Next-generation sequencing technology has been employed to address various biological problems [13], such as muscle development [14], environmental stresses [15,16] and disease resistance [17]. Several studies have identified that ncRNAs stimulate muscle growth and proliferation by modulating the expression of key animal genes, such as miRNAs [14,18], lncRNAs [19], and piRNAs [20]. Transcriptome sequencing is a powerful technique that can identify the intrinsic genetic mechanisms in animals. Abalone is a crucial mariculture mollusk in China. The Pacific abalone *Haliotis discus hannai* (*H. discus hannai*) is known for its high market value and delicate flavor and is the most frequently cultivated abalone in Japan, China, and South Korea [21]. In China, the large-scale aquaculture of *H. discus hannai* was initiated in the late 1980s [21,22]. Abalone farming has spread gradually from the northern Yellow Sea to the East China Sea [23]. In China, about 80% (112,611 tons/139,697 tons) of all abalone is produced in Fujian province [24]. The time required to culture abalone to market size is the predominant factor affecting its production efficiency [25]. The foot muscle is the edible portion of abalone; therefore, many genetic improvement strategies to enhance muscle growth and growth speed have been developed and employed [26]. For efficient selective breeding, it is crucial to investigate the genetic mechanisms associated with muscle growth in *H. discus hannai*. Similarly, research on circRNAs is essential to understand their biological activities. Unfortunately, little is known about the association of circRNA with *H. discus hannai*’s muscle growth.

This research study elucidated the circRNA profiles of the *H. discus hannai* muscle via an Illumina HiSeq 2500 platform. Furthermore, muscle growth-related differentially expressed circRNAs (DE-circRNAs) were identified and their molecular properties, potential functions, and miRNAs binding sites were also evaluated. This research provides the first circRNA data of Pacific abalone and highlights novel molecular pathways of abalone muscle growth.

## 2. Materials and Methods

### 2.1. Animal Materials

*H. discus hannai* was bred at Fuda Aquaculture in Jinjiang, Fujian Province, China. Subsequently, six adductor muscle tissues of *H. discus hannai* were dissected, rapidly snap-frozen in liquid nitrogen, and placed at −80 °C. All specimens were about 2 years old. Three of the samples were larger (“L_HD” group; mean weight, 95.1 ± 7.7 g), and three were smaller (“S_HD” group; mean weight, 16.5 ± 1.0 g) [27].

### 2.2. RNA Extraction and Sequencing

The adductor muscle samples were then employed for RNA extraction and Illumina deep sequencing. Briefly, total tissue RNA was extracted via TRIzol reagent (Invitrogen, Carlsbad, CA, USA), subjected to purity and integrity analyses, and then employed (3 µg/sample) in generating a cDNA library. The TruSeq PE Cluster Kit v3-cBot-HS with the cBot Cluster Generation System (Illumina, San Diego, CA, USA) was utilized to cluster the index-coded sample. For sequencing libraries, an Illumina HiseqX platform was utilized. Then, 150 base paired-end reads were produced. CircRNA libraries of adductor muscle tissues of *H. discus hannai* were constructed using mRNA-seq methods, except that linear RNA digested by 3U of RNase R (Epicentre, Madison, WI, USA) was added before assessment. For quality control, raw data were firstly prepared in fastq format to remove reads containing adapter contaminants and poly-N. Q20, Q30, and GC scores were then calculated using the clean data. RNA-seq data have been submitted to the NCBI.

### 2.3. Identification and Analysis of circRNA

**Figure 1:**
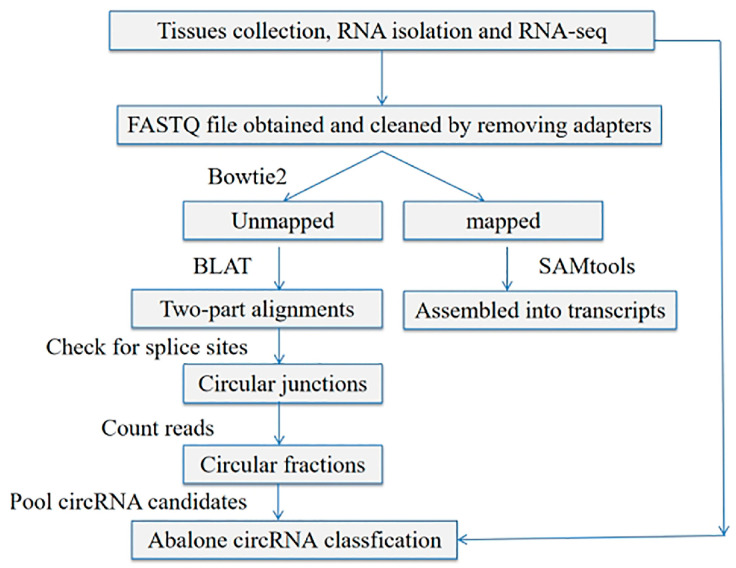
The workflow of identifying circRNA.

The acquired reads were mapped to the reference genome of *H. discus hannai* (the files provided by Dr. Weiwei You, Xiamen University, Xiamen) via Bowtie2 [28] and then filtered using the SAMtools [29]. Furthermore, the find_circ package was employed to identify circRNA [30]. The sequence structure and distribution of circRNA in abalone muscle were analyzed statistically by reference to genome comparison results, and the basic characteristics of circRNA were analyzed. Figure 1 indicates the study workflow. The differential expression assessment of circRNAs was carried out via the DESeq2 package [31]. Adjusted p-values were calculated to take into account the false discovery rate. The read counts of circRNAs were first normalized using transcripts per kilobase million (TPM). Transcripts showing fold changes ≥ 2, with q-values ≤ 0.05, were classified as differentially expressed. Furthermore, GO and KEGG functional enrichment analyses of significantly differential circRNAs were carried out. The GOseq enrichment analysis method was used. Differentially expressed circRNAs (DE-circRNAs) between the two groups were identified by *p* < 0.05 and log2 (fold change) > 1.

### 2.4. Construction and Analysis of ceRNA Networks

To elucidate circRNA-miRNA-mRNA interactions, the ceRNA theory was employed to establish networks [32]. In total, 2267 DE-mRNA and 10 DE-miRNA sequences were obtained from Huang et al. [27,33]. Moreover, miRanda and psRobot were utilized to distinguish circRNA binding sites for miRNAs. Further, the networks were graphically presented with the help of Cytoscape software (version 3.5.1) [34].

### 2.5. Real-Time Quantitative Reverse Transcription PCR (qRT-PCR) Verification

The qRT-PCR test was carried out to assess gene expression levels using specific primers (Table 1). The relative gene expression were normalized using *β-actin* expression. The CFX96 Real-Time System (BIO-RAD, Hercules, CA, USA) was employed for PCR amplification using 25 μL reaction mixtures [2× M5 HiPer SYBR Premix EsTaq (withTli RNaseH; 10 μL), cDNA (2 μL; 100-fold dilution), forward and reverse primers (0.5 μL; 10 μM each), and distilled water (9.5 μL)]. The PCR parameters were as follows: 30 S of 95 °C, 40 cycles of 95 °C for 5 s, and 60 °C for 30 s. At the end of each cycle, the fluorescent signal intensities were assessed. Relative gene expression levels were quantified using the 2^−∆∆CT^ method [35]. Three independent biological replicates were performed. All of the measurements were made in triplicate.

## 3. Results

### 3.1. Characteristics of circRNAs

**Figure 2:**
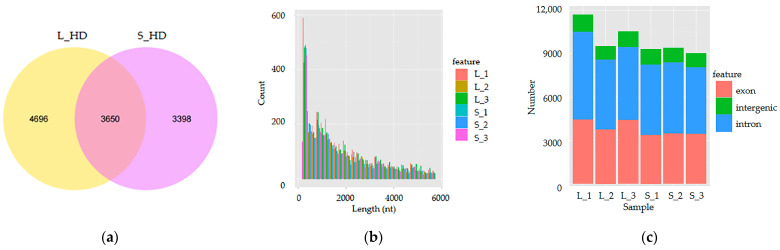
Identification and features of circRNAs. (**a**) The Venn diagram of circRNAs. (**b**) The length distribution of most circRNAs. (**c**) The genomic location of circRNAs.

This research study generated 709,386,602 raw RNA-seq reads (NCBI accession no. SRP126378) and 688,261,544 clean reads remained. We were able to map between 64.09% and 68.95% of the clean reads in each library to the *H. discus hannai* reference genome. We identified 11,744 novel adductor muscle circRNAs (Table S1). Of these, 4696 and 3398 circRNAs were uniquely expressed at L_HD and S_HD group abalones, respectively, while 3650 circRNAs were expressed in both groups (Figure 2a). The average, maximum, minimum, and median sequence lengths of these circRNAs were 4854.6, 95,659, 150, and 1434 nt, respectively. Furthermore, most circRNAs were 150 to 6000 nt long (Figure 2b). Moreover, circRNAs mostly comprised exon (38.70%) and intron (50.91%), with only a small number of intergenic regions (10.39%; Figure 2c).

### 3.2. Differentially Expressed circRNAs and Functional Analysis

**Figure 3:**
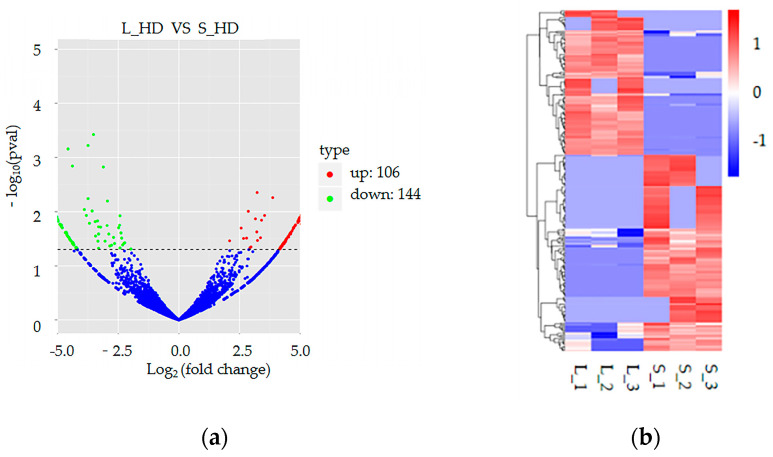
Heatmaps and volcano plots of differentially expressed circRNAs (DE-circRNAs). (**a**) CircRNA expression in large (L_HD) vs. small (S_HD) abalone group. Red and green dots indicate up- and downregulated circRNAs, respectively. (**b**) Hierarchical clustering of DE-circRNAs. Red rectangles represent upregulated circRNAs; blue rectangles represent downregulated circRNAs.

DE-circRNAs are shown in Figure 3, and their details are indicated in Table S2. The data indicated 250 significant DE-circRNAs between the S_HD and L_HD groups. Compared to the S_HD group, 106 and 144 DE-circRNAs were up- and downregulated, respectively, in the L_HD group (Figure 3a). Furthermore, the heat maps revealed that circRNAs had a significant expression difference between the two groups (Figure 3b).

The GO biological process of the host DE-circRNA genes included lipid transport, lipid localization, lipid biosynthetic process, single-organism metabolic process, etc. (Table S3). Furthermore, GO molecular function indicated that the host DE-circRNA genes were enriched in lipid transporter activity, ATP binding, catalytic activity, adenyl ribonucleotide binding, etc. (Table 2). KEGG pathways of DE-circRNAs were enriched in fat digestion and absorption, hypertrophic cardiomyopathy (HCM), dilated cardiomyopathy, vitamin digestion and absorption, tight junction, thyroid hormone signaling pathway, hippo signaling pathway, etc. (Table 3). KEGG enrichment results indicated these DE-circRNAs might be associated with growth-related pathways.

### 3.3. CircRNA-miRNA-mRNA Interaction Network Analysis

**Figure 4:**
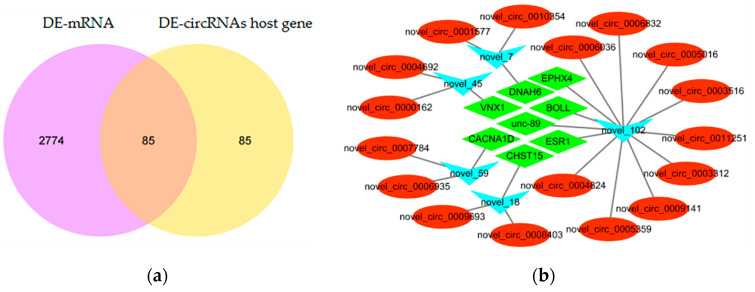
Competitive endogenous RNA (ceRNA) network of circRNA-miRNA-mRNA interactions in the S_HD and L_HD groups. (**a**) Relationship between DE-circRNA and DE-mRNA. (**b**) The interaction between miRNA and circRNAs. The miRNAs are shown in blue. The mRNAs are represented in green. The circRNAs are represented in red.

To elucidate whether abalone circRNAs modulate gene transcription by interacting with miRNAs, circRNAs’ miRNA binding sites were predicted via miRanda and psRobot. An interaction was observed between DE-circRNAs and predicted miRNA targets. The acquired DEcircRNA–DEmiRNA–DEmRNA interaction network indicated nodes and connections between circRNAs, target miRNAs, and mRNA. Furthermore, there were 85 overlapping genes between the host DE-circRNA genes and DE-mRNAs (Figure 4a and Table S4). Moreover, there were no miRNAs regulated by only one circRNA (Figure 4b, Tables S5 and S6). For example, miRNA novel_102 is associated with the regulatory pathway for circRNA novel_circ_0009141, circRNA novel_circ_0005016, etc. In total, five miRNAs, 17 circRNAs, and eight circRNA host genes were involved in the network (Figure 4b). These data indicated that circRNAs could modulate gene expression by acting as miRNA sponges. Furthermore, each circRNA can comprise more than one miRNA interaction site.

### 3.4. Verification by qRT-PCR

**Figure 5:**
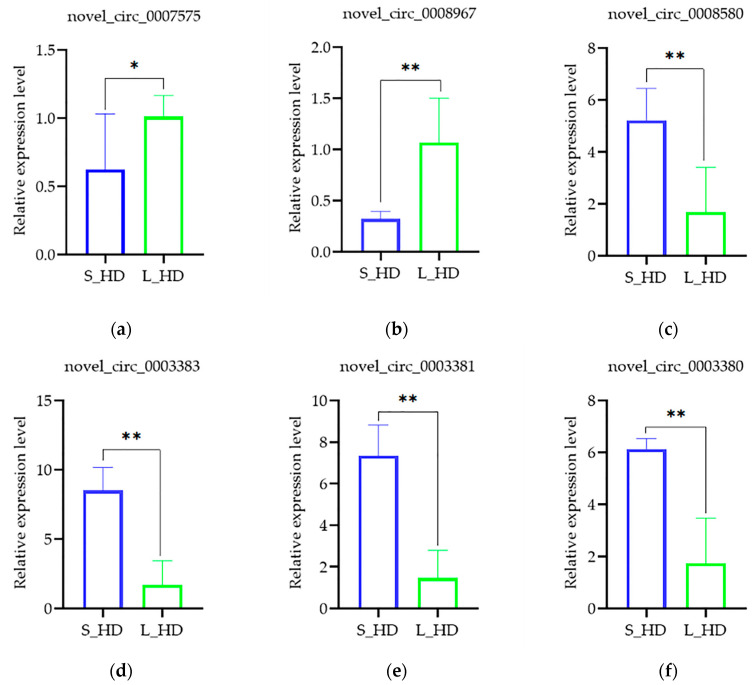
Relative expression of DE-circRNAs quantified with qRT-PCR. (**a**–**f**) The relative expression levels of novel_circ_0007575, novel_circ_0008967, novel_circ_0008580, novel_circ_0003383, novel_circ_0003381, and novel_circ_0003380, respectively. Data are shown as mean ± SD (n = 3). *, *p* < 0.05; **, *p* < 0.01.

The transcript expression was verified by qRT-PCR. To validate the acquired sequencing results, two upregulated DE-circRNAs (novel_circ_0007575 and novel_circ_0008967) and four downregulated DE-circRNAs (novel_circ_0008580, novel_circ_0003383, novel_circ_0003381, and novel_circ_0003380) were selected for qRT-PCR (Figure 5). The expression trends of these DE-circRNAs were consistent with the sequencing data, indicating the accuracy of the acquired RNA-seq data.

## 4. Discussion

The discovery of circRNAs has invigorated the field of RNA research. Their depth, breadth, conservatism, specificity, and stability indicate that they play significant roles in various biological processes [36]. Comprehensive research studies have revealed their functions, such as the interaction between circRNA and animal growth and development. For example, circ-ZNF609 has been observed to be highly expressed in myoblasts, where it promotes myoblast proliferation [37]. Nedoluzhko et al. [38] revealed that in Nile tilapia (*Oreochromis niloticus*), many circRNA genes were associated with muscle function and myogenesis. Abalone growth is controlled by numerous factors as well as transcriptional regulation. Currently, research studies on the function of circRNA in abalone growth are limited. Here, RNA-Seq technology was employed to assess the muscle growth-related circRNA in *H. discus hannai*. To the best of our knowledge, this is the first report to profile circRNA expression in *H. discus hannai* muscle. The data revealed 11,744 novel circRNAs in the *H. discus hannai* muscle, of which 4696 and 3398 circRNAs were expressed in L_HD and S_HD groups. Moreover, these circRNAs mostly comprised exon (38.70%) and intron (50.91%) circRNAs with a small number of intergenic regions. Overall, it was inferred that most abalone circRNAs come from the CDS and intron regions, which was consistent with other species [39], validating the suitability and reliability of the reads acquired from *H. discus hannai* for further analysis.

In vertebrates, muscles constitute 30–80% of the body mass and are involved in metabolism, locomotion, and homeostasis [40]. Muscle growth and development is a complex life activity regulated by a complex network of transcription factors, genes, signaling pathways, ncRNAs, and epigenetic modifications [41,42,43]. Several studies have identified various circRNAs in muscles of different vertebrate taxa, such as birds, teleosts, and mammals [44,45,46,47]. The estimated number of circRNAs in skeletal and cardiac muscle ranges between 622 and 38,000 [11]. Their varied number in different species indicates their dynamic expression in muscle. It has been observed that muscle circRNAs are primarily derived from exons, suggesting their modulatory role at the post-transcriptional level [45]. Moreover, the circRNAs’ functional clustering revealed that they are related to various essential signaling pathways, including *Wnt*/*PI3K-Akt*, which is crucial for muscle development [45]. In this investigation, we analyzed the circRNA expression profile in the muscle in comparing the L_HD and S_HD abalones to assess the potential role of circRNA in *H. discus hannai*’s muscle growth. The data indicated that in the L_HD group, 144 and 106 circRNAs were downregulated and upregulated, respectively compared to the S_HD group. These data revealed that the 250 DE-circRNAs might be associated with growth. The GO terms of the host DE-circRNA genes were associated with many biological functions, such as lipid transporter activity, catalytic activity, ATP binding, adenyl ribonucleotide binding, etc. These results indicated that fast-growth abalone (L_HD abalones) may uptake adequate energy (e.g., ATP, lipid, and nucleotide) from fodder, with excess energy substances employed to increase muscle growth after daily life activities are maintained. Therefore, improving food intake might be essential for increasing slow-growth (S_HD abalones) abalone growth.

In addition, the enriched KEGG pathways included HCM, vitamin digestion and absorption, dilated cardiomyopathy, fat digestion and absorption, tight junction, the thyroid hormone signaling pathway, and the hippo signaling pathway. Previous studies have shown that tight junction proteins participate in the regulation of cell proliferation, gene expression, and cell differentiation [48,49]. Thyroid hormones are crucial for organismal development and homeostasis [50]. The growth of an organism depends on its absorptive and digestive capabilities of animals. Efficient digestion and absorption of dietary fat are crucial to infant growth and development [51]. Moreover, studies have indicated that curcumin is beneficial for the growth performance of fish by improving intestinal growth and development, intestinal digestion, and absorption, and amino acid transportation abilities [52]. Dietary vitamin C requirements for growth and development of fish [53]. The hippo pathway is the main modulator of tissue growth and is an evolutionarily conserved signaling cascade associated with various biological pathways, such as organ size control, cell growth, and regeneration [54]. The genes (*Actb*, *Actc*, *unc-89*, *ESR1*, *tropomyosin*, *MEGF10*, *HDAC4*, etc.) involved in the KEGG pathway were associated with muscle growth [55,56,57,58,59]. All these results indicate that these DE-circRNAs might affect muscle growth in *H. discus hannai*.

Muscle development is a complex system modulated by various factors including circRNAs. Studies have indicated that circRNAs bind miRNAs, thereby regulating gene expression at the post-transcriptional level [28]. CDR1as acts as a miRNA sponge that interacts with miR-7 and helps mRNAs escape degradation after miRNA binding [30,60]. *Sry* can form a circRNA and act as a miRNA sponge [60]. The research suggests that circRNA9210-miR-23a-*MEF2C* and circRNA290-miR27b-*Fox3* are competing endogenous networks in skeletal muscles, which are associated with muscle fiber-type switching [11]. CircSNX29 acts as a ceRNA and its overexpression sequesters miR-744 away from *Wnt5a* to enhance *Wnt5a* levels and *PKC* phosphorylation, thus activating the *Wnt5a*/*Ca2+*/*CaMK-IIδ* pathway and promoting myoblast differentiation [61]. To evaluate if abalone circRNAs modulate gene transcription by interacting with miRNAs, the association between DE-circRNAs and predicted target miRNAs was analyzed. No miRNAs were found to be regulated by only one circRNA, consistent with the study of Liang et al. (2017) [28]. Here, it was observed that 5 miRNAs, 17 circRNAs, and 8 circRNA host genes constituted the network (Figure 5b). These data indicated that abalone circRNAs could also modulate gene expression by acting as miRNA sponges. The 8 circRNA host genes included voltage-dependent calcium channel type D subunit α-1 (*CACNA1D*), protein boule-like (*BOLL*), muscle M-line assembly protein unc-89 (*unc-89*), low-affinity vacuolar monovalent cation/H(+) antiporter (*VNX1*), estrogen receptor (*ESR1*), epoxide hydrolase 4 (*EPHX4*), dynein heavy chain 6 (*DNAH6*), and carbohydrate sulfotransferase 15 (*CHST15*). The literature has indicated the significance of these genes in cell growth and development. For example, *BOLL* can rescue the progression of fly meiotic divisions [62]. Kuales et al. (2011) indicated the involvement of BOLL genes in male and female gamete development in an organism [62]. Furthermore, *ESR* has been observed to promote cell growth and survival by altering gene transcription [56]. The *unc-89* plays an important role in maintaining the structure of muscle cells [55]. A normal structure of muscle cells is the foundation for efficient and effective muscle contraction [55]. Absent or aberrant *unc-89* may cause disruptions in the structure of muscle cells, which could affect muscle growth and development [55]. Therefore, we hypothesized that novel_circ_0006036 and novel_circ_0004824 competitively bind to miRNA novel_102 in conjunction with *unc-89*, thereby reducing the concentration of miRNA novel_102. As a result, the inhibitory effect of miRNA novel_102 on *unc-89* is diminished, and this effect may be achieved through hypertrophic cardiomyopathy (HCM) and dilated cardiomyopathy. Altogether it was observed that the abalone DE-circRNAs may be involved in abalone growth by functioning as miRNA sponges. In this study, we employed transcriptome and bioinformatics techniques to identify DE-circRNAs associated with abalone growth, as well as their interaction relationships with mRNAs and microRNAs. However, several limitations remain. For instance, validation was not performed on a large number of diverse *H. discus hannai* samples, and no relevant experiments were conducted to explore the genetic status of these circRNAs in the offspring. In future, we aim to expand the abalone population and further investigate the functional roles of abalone circRNAs.

## 5. Conclusions

In summary, this study evaluated the circRNA expression profiles of *H. discus hannai* via Illumina HiSeqX sequencing and identified 11744 circRNAs. Furthermore, 250 DE-circRNAs were identified in L_HD and S_HD *H. discus hannai* samples. Moreover, it was observed that circRNAs act as miRNA sponges and regulate gene transcription by interacting with miRNAs. Further, many DE-circRNAs were found to influence *H. discus hannai’s* muscle growth via circRNA-miRNA-mRNA interactions. This research study furnishes comprehensive data on the biological mechanisms controlling abalone muscle growth. To the best of our knowledge, this study is the first to investigate circRNAs in *H. discus hannai*. The findings provide a basis for future research aimed at exploring the molecular functions of abalone circRNAs. These data provide a reference for improving the efficiency of artificial selection and contribute to the genetic improvement of the abalone aquaculture industry.

## Figures and Tables

**Table 1:** Sequences of the primer pairs used in this study.

Primer	Sequence (5′-3′)
novel_circ_0007575-qF	GGAGACAAAGTTGACGGGAT
novel_circ_0007575-qR	AGCACGACATTTGTACGCAG
novel_circ_0008967-qF	AGAGGTGGCATCAGGATCAG
novel_circ_0008967-qR	GGGTAGCCATCGATGAGGAA
novel_circ_0003381-qF	AGAACAAGGTCTCCAGCACA
novel_circ_0003381-qR	TCACTTGTCCCACTAAGCCA
novel_circ_0003383-qF	TTGCTGGAAATTGGTGGTCG
novel_circ_0003383-qR	CTTGGAAGCAGACGTCAAGG
novel_circ_0008580-qF	CTGAAACACTGAAACGTATGGAA
novel_circ_0008580-qR	TCAGTGGATGTAATTATCGCGT
novel_circ_0003380-qF	TTGAGATCAGACAGCCAGGG
novel_circ_0003380-qR	TGATGGGGTTACTCTTGCCA
*β-actin*-qF	GGTATCCTCACCCTCAAGT
*β-actin*-qR	GGGTCATCTTTTCACGGTTG

**Table 2:** Top 10 enrichments of the gene ontology (GO) analysis of the host DE-circRNA genes.

Description	Term_Type	*p* Value
Lipid transporter activity	molecular_function	0.0021589
Lipid transport	biological_process	0.0021775
Lipid localization	biological_process	0.0021775
Catalytic activity	molecular_function	0.0037282
Lipid biosynthetic process	biological_process	0.0041697
ATP binding	molecular_function	0.004655
Adenyl ribonucleotide binding	molecular_function	0.004655
Hydrolase activity	molecular_function	0.0050891
Adenyl nucleotide binding	molecular_function	0.00539
Single-organism metabolic process	biological_process	0.005411

**Table 3:** The KEGG functional enrichment analysis of the host DE-circRNA genes in the L_HD and S_HD groups.

MapTitle	*p* Value	Gene
Hypertrophic cardiomyopathy (HCM)	0.0000976	Actin, cytoplasmic 1 (Actb), actin, cytoplasmic(Actc), Twitchin (unc-22), muscle M-line assembly protein unc-89 (unc-89), tropomyosin, Fc receptor-like protein 3 (FCRL3)
Dilated cardiomyopathy	0.000131112	Actb, Actc, unc-22, unc-89, Tropomyosin, FCRL3
Tight junction	0.000852074	Janus kinase and microtubule-interacting protein 3 (Jakmip3), Actb, Actc, Janus kinase and microtubule-interacting protein 3 (Jakmip3), circularly permutated Ras protein 1 (Cpras1), band 4.1-like protein 5 (Epb41l5)
Vibrio cholerae infection	0.001678884	Actb, Actc, V-type proton ATPase catalytic subunit A (Vha68-2), E3 ubiquitin-protein ligase (KCMF1)
Viral myocarditis	0.013475154	Actb, Actc, inter-α-trypsin inhibitor heavy chain H4 (ITIH4)
Thyroid hormone signaling pathway	0.013793361	Actb, Actc, estrogen receptor(ESR1), Cpras1, Golgi integral membrane protein 4 (Golim4)
Adherens junction	0.014536358	Actb, Actc, receptor-type tyrosine-protein phosphatase kappa (PTPRK), multiple epidermal growth factor-like domains protein 10 (MEGF10)
Fat digestion and absorption	0.015652512	Apolipophorins
Vitamin digestion and absorption	0.017022673	Deleted in malignant brain tumors 1 (Dmbt1), apolipophorins
Collecting duct acid secretion	0.020650989	KCMF1
Arrhythmogenic right ventricular cardiomyopathy (ARVC)	0.025900718	Actb, Actc
Synaptic vesicle cycle	0.027482768	KCMF1
Hippo signaling pathway	0.03606938	Actb, Actc, Leucine-rich repeat and calponin homology domain-containing protein 3 (Lrch3)
Pathogenic Escherichia coli infection	0.038107063	Actb, Actc, tubulin β chain
Alcoholism	0.046909914	Glutamate receptor ionotropic, NMDA 2B (Grin2b), Guanine nucleotide-binding protein subunit β, histone deacetylase 4(HDAC4), Cpras1

## Data Availability

The data presented in this study are available upon request from the corresponding authors.

## Supplementary Materials

The following supporting information can be downloaded at https://www.mdpi.com/article/10.3390/genes16010065/s1, Table S1: All the novel circRNAs in *H. discus hannai* adductor muscle; Table S2: All the DE-circRNAs in *H. discus hannai* adductor muscle; Table S3: GO biological process of the host DE-circRNA genes in *H. discus hannai* adductor muscle; Table S4: Overlapping genes between the host DE-circRNA genes and DE-mRNAs in *H. discus hannai* adductor muscle; Table S5: DEcircRNA–DEmiRNA–DEmRNA interaction network of *H. discus hannai*; Table S6: The mature_sequence of DEmiRNA from *H. discus hannai*.
